# Development of an intelligent food nutrition recognition and nutrient intake assessment system in hospital settings

**DOI:** 10.3389/fnut.2026.1637034

**Published:** 2026-04-28

**Authors:** Boshi Wang, Chenyu Nong, Jiayu Zhang, Jing Lv, Shilong Zhao, Longxiang Li, Peng Liu

**Affiliations:** 1 Department of Clinical Nutrition, Peking University People's Hospital, Beijing, China; 2 Department of Food and Nutrition, Macau University of Science and Technology, Macau, China

**Keywords:** artificial intelligence, clinical nutrition, dietary survey, nutrient intake, segment anything model

## Abstract

**Background:**

Hospital nutritional diets significantly impact healthcare service standards, therapeutic outcomes, and patient satisfaction. Traditional manual dietary survey methods suffer from substantial limitations and high resource demands. Despite technological advances, few systems have successfully integrated machine vision, electronic weighing, and standardized databases for real-time nutrient assessment in clinical settings.

**Objective:**

To develop and validate an intelligent food nutrition recognition and nutrient intake assessment system for hospital therapeutic diets, enabling precise, large-scale monitoring of patient nutrient intake.

**Methods:**

We developed an AI-based image recognition system integrating RGB-D imaging, the Segment Anything Model (SAM) for food image segmentation, and the SE_ResNet50_vd model for food classification. The system comprised hardware components (food weight collector, image collector, computing host) and software modules (nutritional analysis platform developed in Java/Spring Boot). A comprehensive database was constructed from 204 standardized therapeutic diet varieties from Peking University People's Hospital, incorporating the Chinese Food Composition Table and cooking loss factors. Post-meal intake was calculated by comparing pre- and post-meal food volumes using depth camera imaging and density-based weight estimation. Food type recognition accuracy was tested on 1,000 samples, and volume estimation accuracy was validated against gravimetric measurements across 100 food items.

**Results:**

The food image recognition model achieved 99.2% accuracy on the test set. Volume estimation accuracy exceeded 90% in 39% of cases and 80–90% in 61% of cases, with a minimum threshold of 80% across all tested items. The SAM model demonstrated robust segmentation performance for diverse food types in standardized meal containers. The integrated system successfully monitored over 20 types of therapeutic diets, matching nutrient compositions to personalized requirements with ≥90% accuracy for meal verification.

**Conclusions:**

This intelligent system provides standardized, real-time nutrient intake assessment with superior accuracy compared to traditional dietary survey methods. By automating therapeutic meal supervision and enabling precision nutrition monitoring, it represents a significant advancement from empirical to precision-based clinical nutrition practice, with substantial potential for improving treatment accuracy, patient outcomes, and healthcare resource optimization in hospital settings.

## 1 Introduction

The quality of hospital nutritional diets significantly impacts healthcare service standards. Providing high-quality nutrition therapy and dining services ensures patients' nutritional needs are met, promotes recovery, and enhances satisfaction. Clinically, hospital therapeutic diets are a vital component of the clinical nutrition diagnosis and treatment system, playing a key role in improving health status, aiding postoperative recovery, and restoring functional capacity.

Current dietary survey methodologies, including the 24-h dietary recall, food frequency questionnaires (FFQ), and weighed food records, exhibit well-documented limitations in clinical and research contexts. Studies demonstrate that self-reported food descriptions achieve only 60–70% accuracy, with significant underestimation of portion sizes and nutrient content. Additionally, these methods are labor-intensive, require highly trained personnel, and impose considerable burden on both patients and healthcare providers. While image-based dietary assessment techniques have emerged as promising alternatives, existing approaches often lack integration with precise weighing systems and standardized nutrient databases, limiting their applicability for large-scale clinical implementation. To address these critical gaps, we developed an artificial intelligence (AI)-based image recognition system that automates therapeutic meal supervision and enables precise, real-time nutrient intake assessment.

The present study describes the design and technical implementation of this intelligent nutrition assessment system within a tertiary hospital setting. We hypothesized that the integration of machine vision, electronic weighing, and standardized therapeutic diet databases would achieve ≥90% accuracy in food recognition and ≥80% precision in volume estimation, thereby providing a robust foundation for evidence-based clinical nutrition management.

## 2 Materials and methods

### 2.1 Materials

A total of 204 varieties of therapeutic diets provided by Peking University People's Hospital for inpatients were selected as the study materials. These foods were standardized during preparation, with image and weight data collected to construct a data set for food and recipes used in both training and testing phases.

The dataset mainly contains two core types of data:

(1) Image data: Standardized images of the 204 types of therapeutic diets mentioned above were collected. These images serve as the raw input data and, after preprocessing steps such as image scaling, cropping, and brightness/contrast adjustment, are used to train image recognition and segmentation models.
(2) Recipe and Nutrition Database: A comprehensive database was constructed to store detailed ingredient information and their corresponding nutrient data, including essential components such as energy, protein, fat, carbohydrates, vitamins, and trace elements. This database is integrated with the recipe database to form a system foundation capable of providing precise nutritional information.

### 2.2 System structure and function

#### 2.2.1 System platform construction

The system comprises hardware and software components. The hardware includes a food weight collector, a food image collector, and a computing host. The computing host primarily utilizes nutritional analysis software for data collection and processing tasks. The software was developed in a Java environment using Spring Boot on the Windows operating system.

#### 2.2.2 System function and operation

Considering existing technology and the hospital application scenario, this design employs a combination of weighing and camera recording to accurately record patients' nutritional intake. Given that the hospital's therapeutic diet recipes and food combinations are relatively fixed, it is feasible to establish a picture database and match the nutrients of each meal. This system effectively addresses the reliance on memory and descriptive abilities in traditional methods while eliminating human error during measurement. The system ensures that the nutrient composition of over 20 types of therapeutic diets aligns with personalized intake requirements.

### 2.3 Methods

#### 2.3.1 Database construction

A comprehensive database was established to store detailed information about food ingredients and their corresponding nutrients, including energy, protein (percentage of high-quality protein), fat (ratios of saturated and unsaturated fatty acids), carbohydrates, vitamins, trace elements, dietary fiber, and other essential components.

#### 2.3.2 Food recognition system

The system integrates an intelligent recognition module to identify food types and quantities, ensuring precise calculation of nutrient intake. This module employs advanced image analysis technology to achieve rapid and accurate identification of foods and their respective portions.

#### 2.3.3 Nutritional analysis process

After patients finish their meals, regardless of whether any food remains uneaten, they are required to take photos of the meal boxes using their smartphones (equipped with depth cameras). By leveraging Paddle AI online models and positioning markers on the meal boxes, the system calculates the volume of remaining food in each compartment. Combined with recorded data on food type, weight, and volume from meal preparation, the system estimates the actual intake weight of each dish (assuming consistent density for each item). The intake data are then matched to the built-in meal database, enabling precise calculation of the actual nutrient intake based on the established nutritional database.

#### 2.3.4 Image preprocessing

The RGB-D images acquired by the food image collector served as the raw input data. These inputs underwent preprocessing steps, including image resizing, cropping, brightness and contrast adjustment, and normalization targeting specific positioning markers. This preprocessing reduced computational complexity and significantly enhanced the efficiency of model training while preserving critical spatial information for subsequent segmentation tasks.

#### 2.3.5 Food image segmentation

The Segment Anything Model (SAM) is a state-of-the-art image segmentation framework comprising a Vision Transformer (ViT)-based encoder and a prompt-guided decoder. Leveraging the meal box area position markers as guidance, SAM demonstrated exceptional capability in segmenting objects not present in its extensive training data set (SA-1B). This model's robustness and versatility stem from its large-scale training strategy, enabling accurate segmentation across diverse scenarios.

The key strength of SAM lies in its ability to handle arbitrary-shaped inputs and perform semantic segmentation on any specified image region. Given these advantages, SAM was selected as the food image segmentation algorithm in this study. By inputting the preprocessed RGB images into the trained SAM model, the system obtained precise segmentation information and pixel-level localization for each food item within the meal box.

The segmentation algorithm workflow designed in this study explicitly supports processing of single photographs containing multiple dishes. Specifically, the input image first undergoes preprocessing, wherein positioning markers on the food container are utilized to determine bounding boxes for individual compartments. Subsequently, the segmentation model generates multiple independent segmentation masks in parallel within the single image based on these bounding box prompts, corresponding to distinct dish types, respectively. This workflow emulates actual hospital meal provisioning scenarios (multiple dishes per meal), circumventing the cumbersome procedure of physically separating multiple dishes prior to photography, thereby enhancing system practicality and operational efficiency.

#### 2.3.6 Food type recognition

For food classification and recognition, this study utilized the SE_ResNet50_vd model from the Paddle industrial-grade open-source library. This model processes annotated food images to confirm food types by leveraging its advanced architecture. The Squeeze-and-Excitation (SE) module within the model employs global average pooling and two fully connected layers to learn channel-wise relationships, re-calibrating feature maps to enhance focus on discriminative features while suppressing irrelevant ones. This mechanism significantly improves classification accuracy.

#### 2.3.7 Volume recognition

In this study, the core algorithm for volume recognition employed an integrated approach combining three-dimensional reconstruction with voxel-based computation. The technical workflow comprised the following key steps:

Depth Image Acquisition and Enhancement. RGB-D images of food items were acquired using a depth camera, and high-resolution point cloud data were generated through super-resolution depth estimation utilizing the DELTAR deep learning model.

Spatial Mapping and Height Extraction. Based on the point cloud data, a convex hull algorithm was applied to compute the transformation matrix T between RGB and depth image coordinate systems. This mapping matrix enabled the projection of depth data onto segmented food regions, thereby obtaining pixel-level precision for food height information.

Three-dimensional reconstruction and volumetric calculation. The acquired three-dimensional spatial data of food items were voxelized to perform 3D reconstruction. The approximate volume V was subsequently derived through quantification of the total voxel count within the reconstructed food geometry.

#### 2.3.8 Food nutrient calculation

A comprehensive nutrient database was developed based on national standards, including the “Chinese Residents' Dietary Nutrient Reference Intakes” and the “Chinese Food Composition Table.” The database incorporated factors such as edible portions and cooking losses to ensure accurate nutritional profiles. It was integrated with a recipe database to form a system capable of providing precise nutrient information for various therapeutic diets.

The intelligent recognition system ensured that the meal quantities and nutrient compositions of over 20 types of therapeutic diets matched the personalized nutrient intake requirements set in the system after standardized ingredient preparation, cooking, and packaging. Before serving, each meal underwent matching; if the match rate for food types and quantities reached ≥90%, it was deemed acceptable; otherwise, the meal was re-prepared. This system guaranteed that patients received meals with accurate nutritional profiles.

Post-consumption, patients' actual food intake was recorded using RGB-D imaging technology combined with SAM-based segmentation and SE_ResNet50_vd classification algorithms.

The detailed calculation methods were as follows:

1. Determination of actual consumed weight

This study employed a computational method integrating pre-meal weight with pre- and post-meal volumetric measurements. Specifically, the weight of each food item in the meal container was recorded during meal distribution. Subsequently, pre-meal and post-meal food images were acquired to estimate the approximate pre-meal volume and post-meal residual volume of each food item, respectively. The actual consumed weight of each food item was then calculated based on the volumetric ratio between pre- and post-meal states and the initially recorded weight. This approach demonstrated superior accuracy compared to density-based calculations, as food density exhibits substantial variability attributable to differences in culinary preparation methods and cooking parameters, resulting in non-constant density values across different meal instances.

2. Algorithm for nutrient intake calculation

The core of nutrient calculation involved the integration of actual consumed weight with a pre-established nutrient database. The system computed actual nutrient intake based on the following procedural steps: (1) comparative analysis of pre- and post-meal food images to determine volumetric reduction ratios and estimate actual consumed food weight; (2) matching against the constructed recipe database to identify specific food types and their respective weights for the individual patient; and (3) automated calculation of nutrient intake for each food category based on nutrient composition data from the database, including energy, protein, fat, carbohydrates, vitamins, minerals, and dietary fiber.

The complete technical pathway from data collection, image preprocessing, segmentation and recognition to final nutrition calculation was organized as follows:

S1 Construct database, including recipe database and nutrient database.
S2 Obtain the weight of various meals in the meal box during meal serving.
S3 Acquire pre-meal food images, identify recipe types based on the recipe database; and estimate food volume based on meal images to obtain approximate pre-meal volume of each food item.
S4 Acquire post-meal food images, estimate food volume based on meal images to obtain approximate post-meal volume of each food item.
S5 Calculate actual consumed weight of each food item based on the approximate volume ratio between pre-meal and post-meal states and the weight obtained in S2.
S6 Calculate actual nutrient intake based on actual consumed food quantity and the nutrient database.

#### 2.3.9 Statistical methods

**Figure 1:**
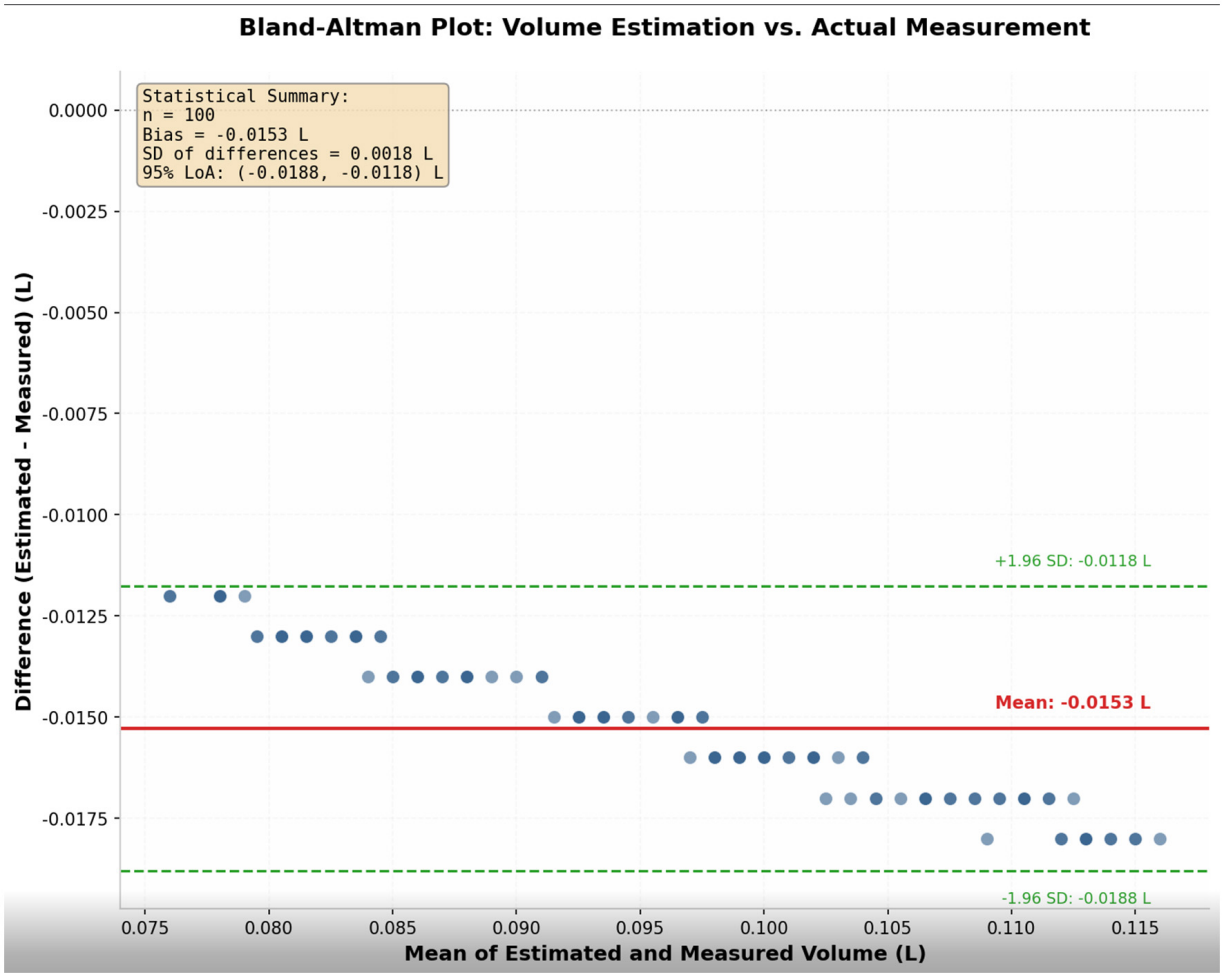
Bland-Altman plot comparing the estimated food volume (V) and measured volume (Vd) across 100 food samples. The mean difference and the 95% limits of agreement are shown.

In our study, we selected 100 different food samples, calculated the ratio between the system-estimated volume (V) and the measured volume (Vd) (*P* = V/Vd), and conducted the following statistical analyses:

(1) Basic Statistics:
  We calculated the mean and standard deviation of this ratio. The results showed that the mean ratio was 85.4%, with a standard deviation of 5.2%.
(2) Bland-Altman Agreement Analysis:
  To evaluate the agreement between the two measurement methods, we plotted a Bland-Altman graph. This graph used the difference between the approximate volume (V) and the measured volume (Vd) as the vertical axis, and the mean of the two as the horizontal axis. The analysis results showed that the mean difference was close to 0, and the vast majority of data points fell within the 95% limits of agreement, indicating good agreement between the system-estimated volume and the standard measured volume (Figure 1).
Correlation Analysis:
  We calculated the correlation coefficient between the approximate volume (V) and the measured volume (Vd).

## 3 Model training and results analysis

### 3.1 Performance of food image recognition model

**Figure 2:**
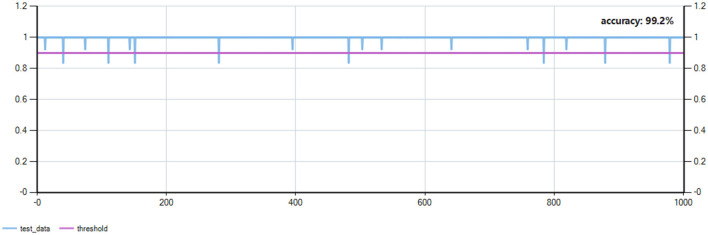
Food image recognition model test set. This chart illustrates the performance of the food image recognition model. The horizontal axis represents the test sample index (ranging from 0 to 1,000), while the vertical axis indicates the prediction confidence or probability, ranging from 0 to 1. The blue line displays the model's prediction confidence for each sample, and the purple horizontal line represents the threshold of 0.8.

The accuracy of food type recognition on the test set (1,000 samples) reached 99.2% (see Figure 2).

### 3.2 Analysis of food volume calculation method

The volume of food, *Vd*, was measured using a gravimetric method by pouring the dish into a standardized measuring cup. This experimentally determined volume served as the reference standard and was compared with the approximate volume calculated in Section 1.3.4. The accuracy of the estimation was defined as *P* = V/Vd.

In assessing volume estimation accuracy across 100 distinct food items, we computed fundamental statistical measures for the ratio (P) of estimated volume (V) to measured volume (Vd), defined as *P* = V/Vd. The results demonstrated a mean ratio of 85.4% with a standard deviation of 5.2%.

**Figure 3:**
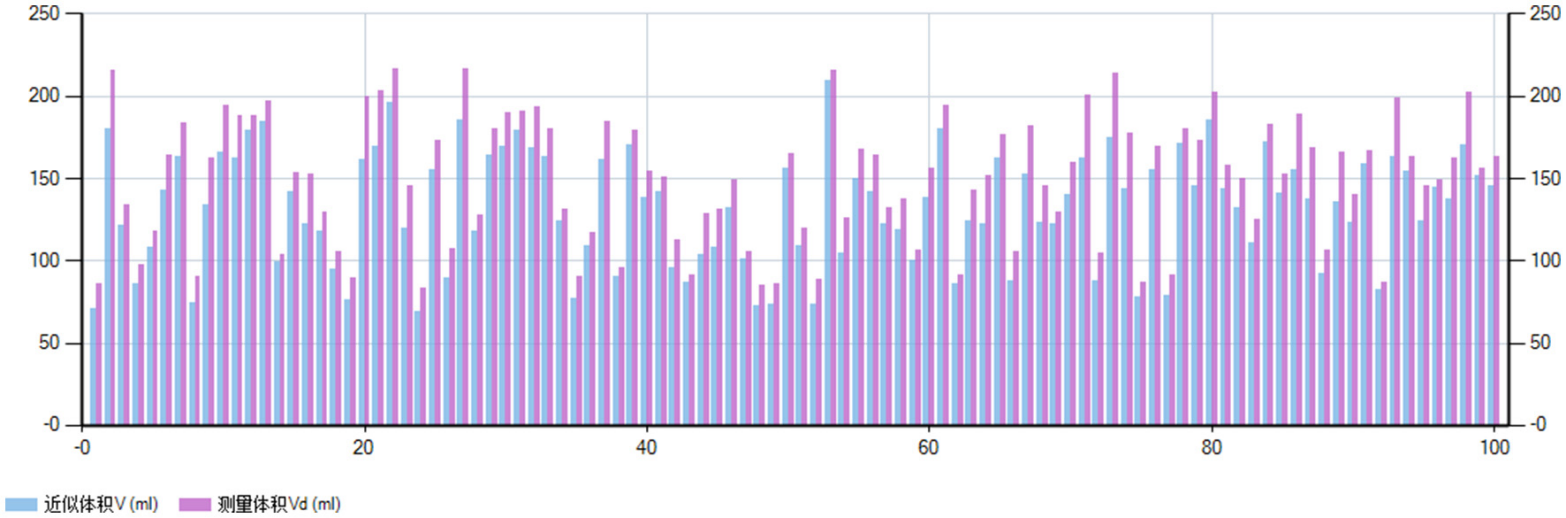
Comparison of approximate volumeand measured volume for 100 different volume foods (ml). This figure compares the approximate volume (V, blue bars) with the measured volume (Vd, purple bars) for 100 different food items, with units in milliliters. The horizontal axis represents the food index (ranging from 0 to 100), while the vertical axis indicates volume, ranging from 0 to 250.

**Figure 4:**
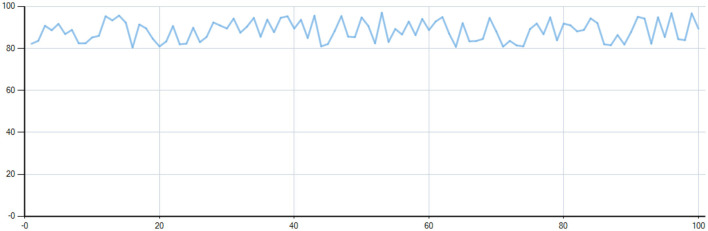
Ratio of approximate volume (V) to measured volume (Vd) for 100 different volume foods. V denotes the “approximate volume” calculated through the system algorithm.

After evaluating 100 different food items across various volumes, the system demonstrated an accuracy greater than 90% in approximately 39% of cases and between 80% and 90% in approximately 61% of cases. These results indicate that the volumetric measurement accuracy achieved a minimum threshold of 80%, with most estimates falling within an acceptable range (as illustrated in Figures 3, 4).

This validation process demonstrates the robustness of the volume estimation method for practical applications in food intake monitoring systems.

### 3.3 SAM model segmentation accuracy example

**Figure 5:**
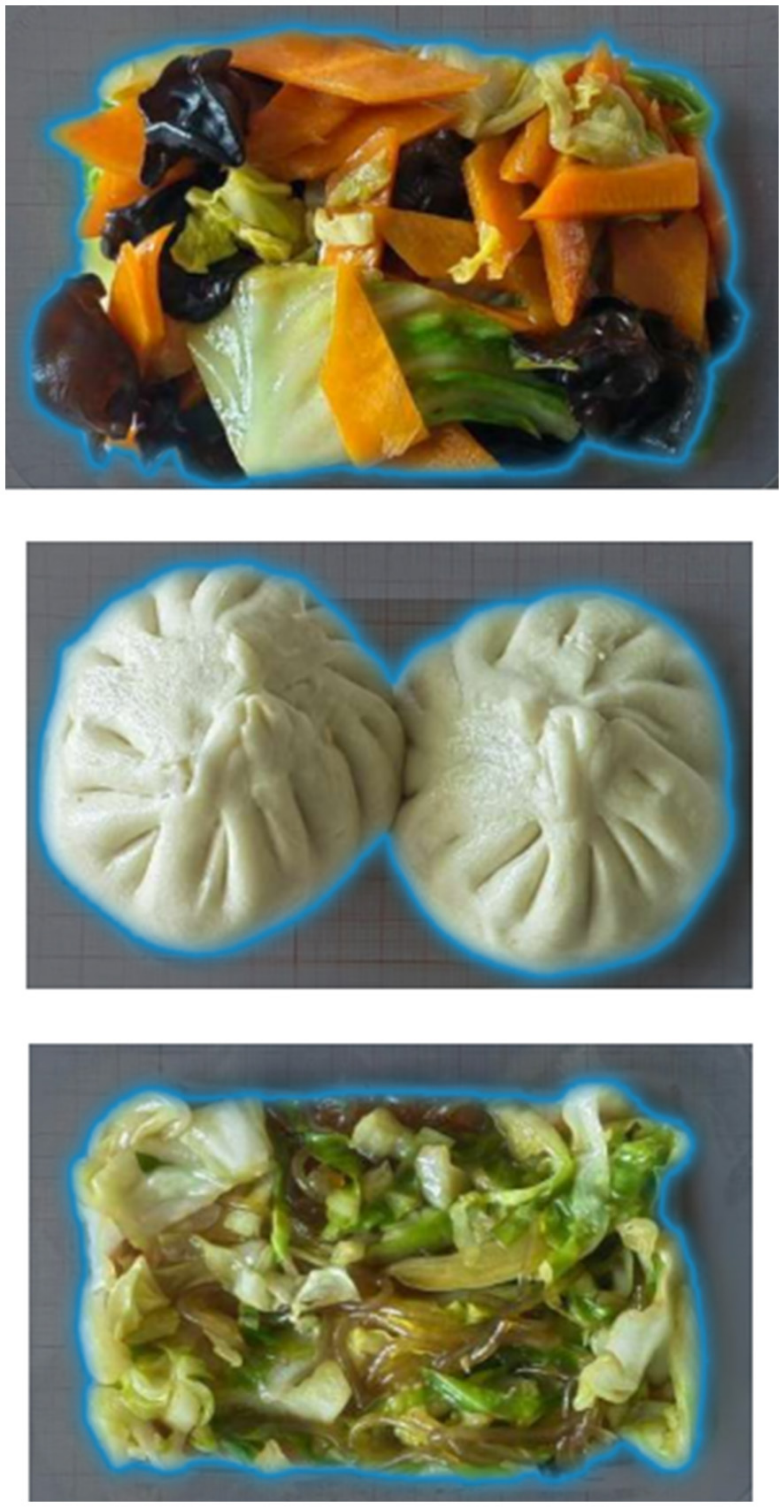
SAM model segmentation accuracy example.

To intuitively demonstrate the segmentation performance of the SAM model in complex scenarios, Figure 5 presents a comparative visualization of the original input image and the generated segmentation masks. As illustrated, for a single image containing multiple dishes (e.g., staple food, side dishes, and soup), the model accurately delineates boundaries between distinct food regions and generates independent segmentation masks. This substantiates that the segmentation strategy employed in this system is based on single photographs encompassing multiple dishes, rather than simplified images of individual food items. Although minor deviations in segmentation edges persist in certain localized regions due to illumination variations and food morphology, the overall segmentation accuracy satisfactorily meets the requirements for subsequent volume calculation and nutritional analysis.

### 3.4 System hardware components

**Figure 6:**
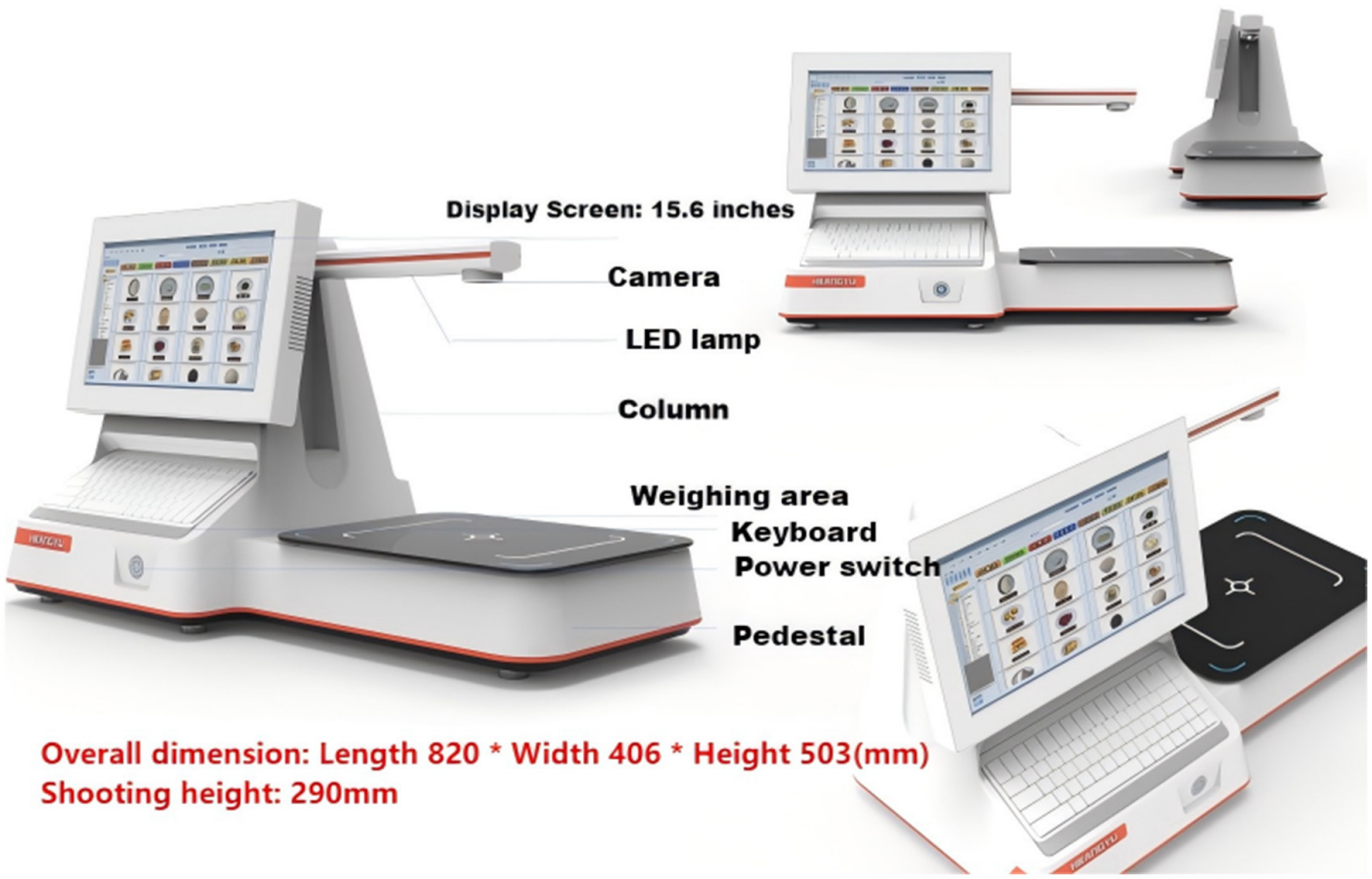
Hardware components of the system platform.

The hardware components mainly include a food weight collector, a food image collector, and a computing host (see Figure 6).

### 3.5 System operation demonstration

**Figure 7:**
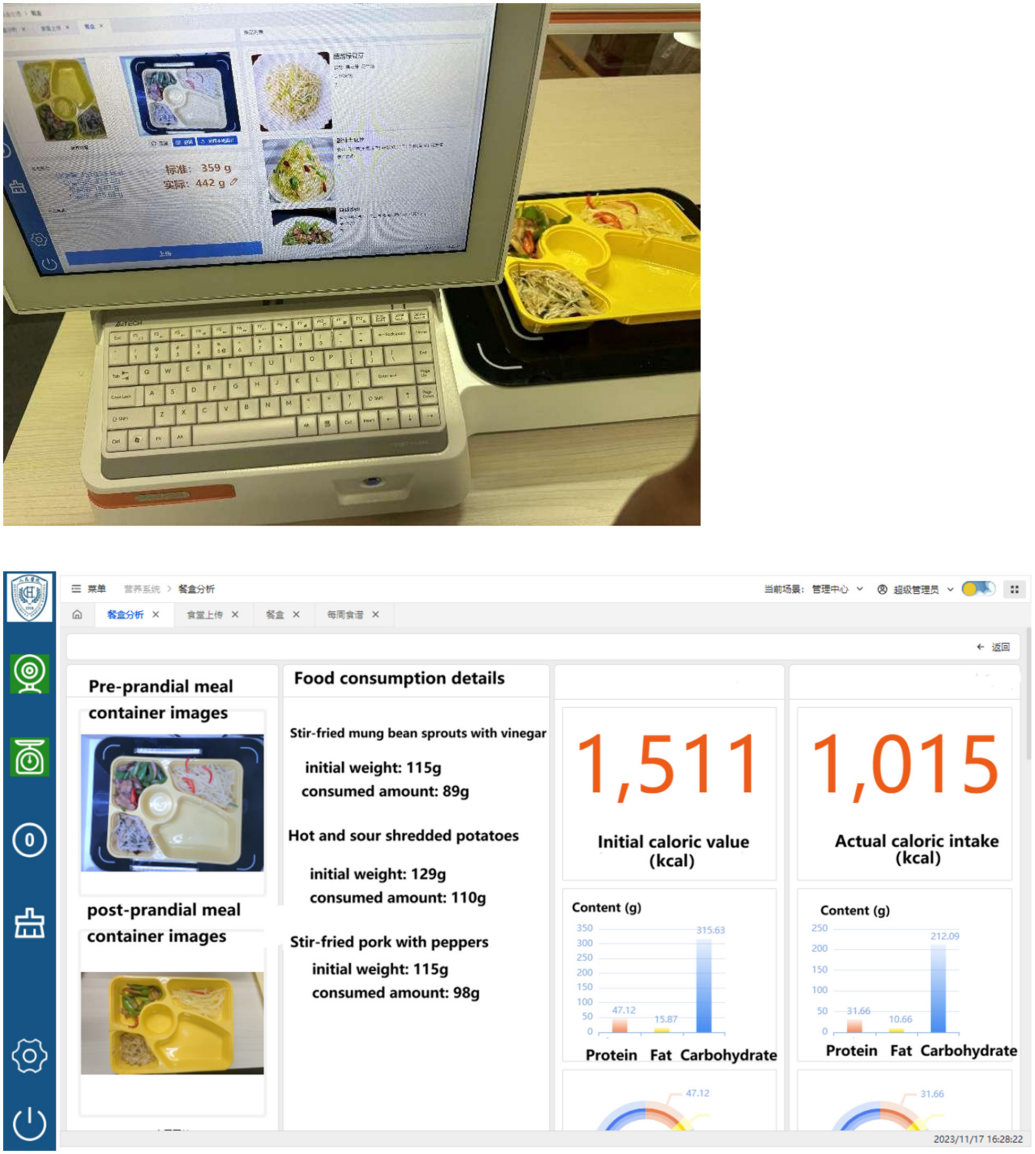
Demonstration of nutrition recognition using photography.

**Figure 8:**
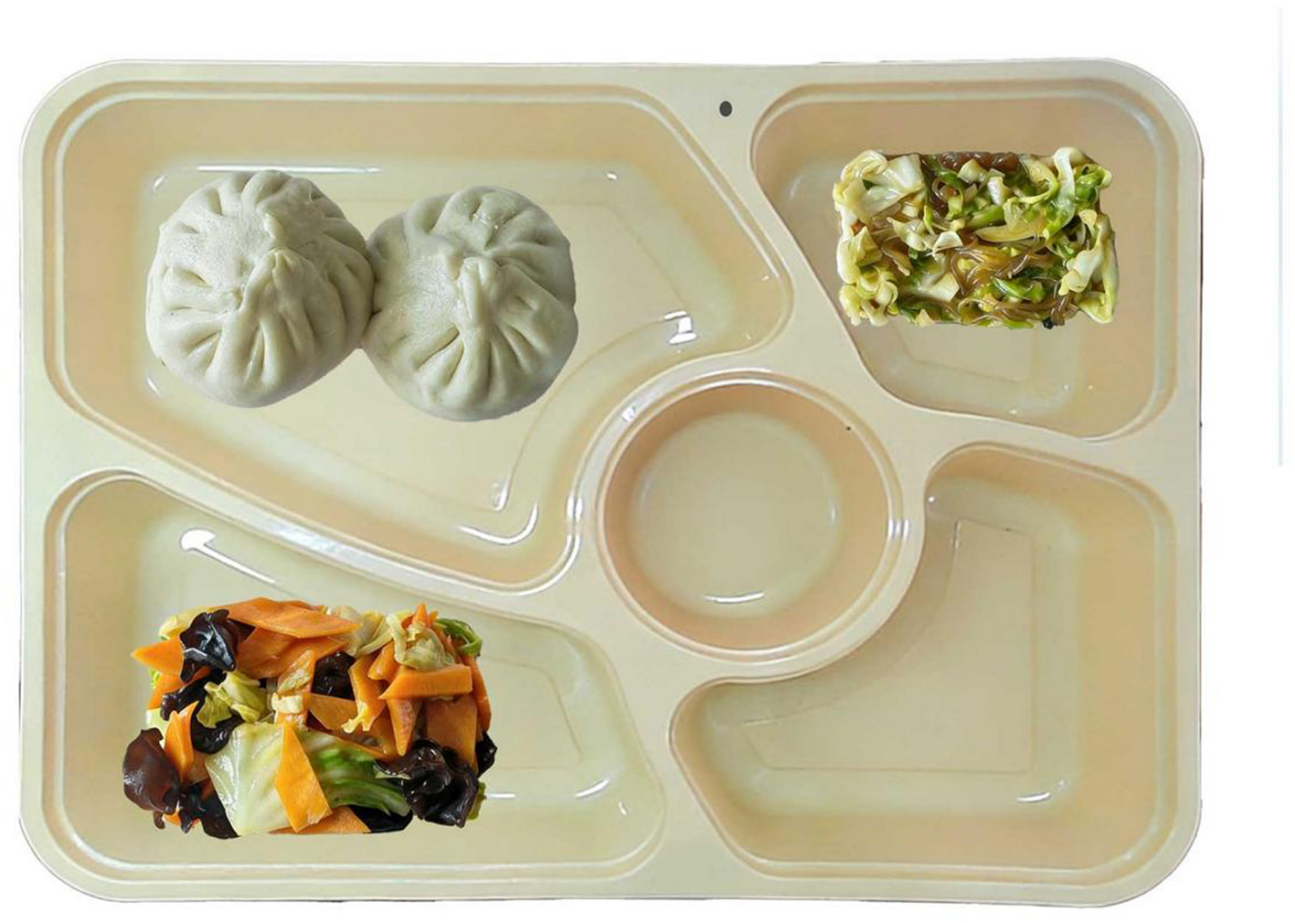
Post-recognition status of the preprandial image following system processing.

The actual operation of the nutrition recognition system using photography is shown in Figure 7. The system interface demonstrates the synchronous recognition and analysis process for meal trays containing multiple dishes (multi-regional), intuitively presenting the recognition results for each food region and the corresponding weight estimation data. Post-recognition status of the preprandial image following system processing is shown in Figure 8.

## 4 Discussion

The intelligent dietary monitoring system designed in this study represents a significant advancement in nutritional research, integrating intelligent recognition systems and electronic weighing technology to address the limitations of traditional dietary survey methods. These conventional approaches, including the 24-h recall method, food frequency questionnaire (FFQ), and food record (FR), have been foundational tools but exhibit notable shortcomings in practical applications.

The 24-h recall method, while widely used, is prone to memory bias, particularly when estimating complex meals. Research indicates that individuals' descriptions of food types and quantities achieve only 60–70% accuracy (1). This limitation is compounded by age-related declines in visual perception, which further impede volume estimation. Additionally, discrepancies between raw and cooked food volumes lead to underestimations of nutrient intake, with errors comparable to the energy content of the foods themselves (2). In China, the reliance on face-to-face interviews increases survey costs and demands higher organizational capacity (17). Investigators must be specially trained to ask provocative questions to respond to the fact that each person's ability to remember, describe and quantify the food they eat varies, and quantitative accounting of intake requires additional time (3).

The FFQ method, which requires subjects to recall dietary habits over extended periods, also presents challenges. The Chinese population's habit of sharing meals, the complexity of traditional dietary composition, and the lack of awareness of the concept of standardized food portion sizes make the measurement of FFQ difficult (4). Memory decay over time leads to significant data distortion, with nutrient intake assessments showing errors as high as 30–40% for vitamins and trace elements (5). Furthermore, under reporting of food intake remains a persistent issue, potentially influenced by participants' tendencies to present healthier dietary habits (6). Additionally the survey takes 30–60 min and respondents may be reluctant to take the survey (7).

Existing methods for food nutrient recognition have demonstrated varying degrees of utility. Claudia et al.'s “food photography 24-h recall method” (FP 24-h) showed promise in rural settings but was limited by its regional applicability and reliance on trained personnel (8). Martin et al.'s (9) remote food photography method (RFPM) provided reliable energy intake estimates but demonstrated significant underestimation across participant groups. Wang Zhixiang's image-based dietary survey technique improved data quality but remained impractical for large-scale applications (10). In addition, there are errors in relying on images alone to determine food intake. Some sauces and soups are difficult to estimate the type and portion size from images, resulting in inaccurate calculation of nutrient intake (11). Taking images to assist with dietary surveys also produces a large number of images, which requires surveyors to have more experience in food identification and nutrient calculations to cope with the increased workload (12).

Despite extensive research, few systems have successfully integrated precise machine vision, electronic weighing, and standardized databases to provide real-time nutrient feedback (13–16). Current nutrient analysis software struggles with complex meals, often relying on user input or fixed recipes rather than truly reflecting individual dietary diversity (17, 18).

The intelligent nutrition assessment system developed in this study addresses these limitations through a multi-modal database combined with real-time image analysis, enabling precise recognition and quantification of diverse food components. This approach not only calculates nutrient content but also dynamically adjusts based on actual consumption patterns.

In application, the system is suitable for both clinical settings and individual use, offering personalized dietary guidance that promotes public health by enhancing awareness of food volume and nutrient composition. This, in turn, contributes to the prevention of chronic diseases such as obesity and diabetes.

Currently, the validation of volume calculation accuracy in this study has been conducted exclusively on pre-meal standardized food samples. Error verification data for post-meal residual food (leftover dishes) volume measurements have not yet been provided.

Although the system design employs identical segmentation and reconstruction algorithms for post-meal volume calculation, post-meal food typically presents complex conditions including morphological alterations, food mixing, and residual liquid. These factors may introduce algorithmic errors in practical applications that differ from pre-meal validation results.

The mean value results indicate that the estimated volumes tend to be smaller than the measured volumes (85.2%). To elucidate this phenomenon, the principal contributing factors were analyzed as follows:

Firstly, Conservative Boundary Segmentation Strategy for Food Containers.

This study utilized standardized food containers equipped with specific positioning markers for image acquisition. During image processing, edge detection and contour analysis techniques were employed in conjunction with positioning markers to obtain bounding box coordinates for various container compartments, which were subsequently used as prompts for the Segment Anything Model (SAM) segmentation. To mitigate the impact of container edge reflections and background interference on segmentation accuracy, the algorithm adopted a relatively conservative approach when processing boundary pixels. This strategy may result in food mask margins that are marginally smaller than the actual food coverage area, thereby introducing systematic underestimation in volume calculations.

Furthermore, Complexity of Food Surface Morphology and Inherent Limitations of Three-Dimensional Reconstruction

The core volume calculation algorithm integrated three-dimensional reconstruction with voxel-based computation. Specifically, acquired three-dimensional spatial data of food items were voxelized to facilitate 3D reconstruction, with approximate volume determined by quantifying intravoxel counts. However, numerous therapeutic diet items (e.g., stir-fried dishes, steamed buns) present surfaces characterized by considerable topographical complexity, including abundant folds, depressions, and irregular undulations rather than smooth curvatures. During 3D reconstruction, these fine-scale surface texture details may be subject to smoothing algorithms, or depth camera imaging limitations regarding specific material properties (e.g., highly reflective or semi-transparent ingredients) may result in incomplete surface height information acquisition. Consequently, estimated volumes tend to be marginally inferior to those obtained through physical water displacement measurement (19, 20).

Finally, Correctability of Systematic Deviation.

Despite the universal underestimation of calculated volumes relative to measured values, statistical analysis revealed an exceptionally small standard deviation for this ratio (0.34%), indicating substantial regularity and consistency in this deviation pattern. Such stable systematic error is amenable to effective correction through the implementation of calibration coefficients or linear regression modeling.

Accordingly, the development of targeted volume correction models based on large-scale sample datasets constitutes a priority direction for our team's forthcoming optimization efforts, with the objective of further enhancing system measurement precision.

Meanwhile, this aspect will serve as the focus of our subsequent research endeavors. We will conduct dedicated clinical trials to perform independent error analysis and validation of volume estimation for post-meal residual food.

Future optimization efforts will focus on several key areas:

- Expanding database coverage to include global recipes and comprehensive nutrient information.
- Improving image recognition algorithms for enhanced accuracy.
- Developing weight estimation functions independent of standard tableware.

Through ongoing innovation and application development, this study aims to advance the field of nutritional science by providing more reliable tools for dietary research.

## Data Availability

The original contributions presented in the study are included in the article/supplementary material, further inquiries can be directed to the corresponding author.
